# Supplementary material for: Structural Property, Immunoreactivity and Gastric Digestion Characteristics of Glycated Parvalbumin from Mandarin Fish (Siniperca chuaisi) during Microwave-Assisted Maillard Reaction
Source: Foods. 2022 Dec 22;12(1):52. doi: 10.3390/foods12010052 (PMC9818276; doi:10.3390/foods12010052)
Supplement: Supplementary file 1 [file foods-12-00052-s001.zip › foods-2024062-Supplementary.pdf]

**Supporting Information**

# Structural Property, Immunoreactivity and Gastric Digestion Characteristics of Glycated Parvalbumin from Mandarin Fish (Siniperca chuaisi) during Microwave-Assisted Maillard Reaction

Jingjing Tai [a,*], Dan Qiao [a,*], Xue Huang [a], Huang Hu [a], Wanzheng Li [a],

Xinle Liang [a], Fuming Zhang[b], Yanbin Lu [a] and Hong Zhang [a]

[a] *School of Food Science and Biotechnology, Zhejiang Gongshang University, Hangzhou 310018, China*

[b] *Department of Chemical and Biological Engineering, Center for Biotechnology and Interdisciplinary Studies, Rensselaer Polytechnic Institute, Troy, NY, 12180, USA*

[c] *Collaborative Innovation Center of Seafood Deep Processing, Key Laboratory of Aquatic Products Processing of Zhejiang Province, Institute of Seafood, Zhejiang Gongshang University, Hangzhou 310012, China*

**Corresponding Author:**

Dr. Yanbin Lu

Phone: +86-571-87103135

E-mail address: luyanbin@zjgsu.edu.cn.

Dr. Hong Zhang

Phone: +86 13858001588

E-mail address: hongzh1316@zjgsu.edu.cn.

*These authors contributed eqully to this work.

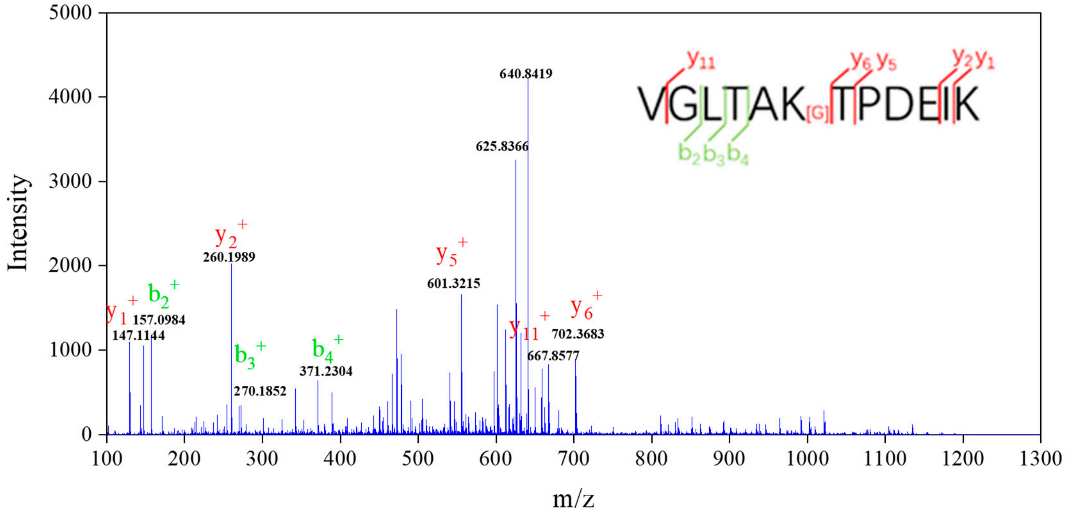
Intensity
m/z
VGLTAK[G]TPDEIK
y11 y6 y5 y2 y1
b2 b3 b4
640.8419
625.8366
y2+
260.1989
y5+
601.3215
y1+
147.1144
b2+
157.0984
b3+
270.1852
b4+
371.2304
y11+
667.8577
y6+
702.3683

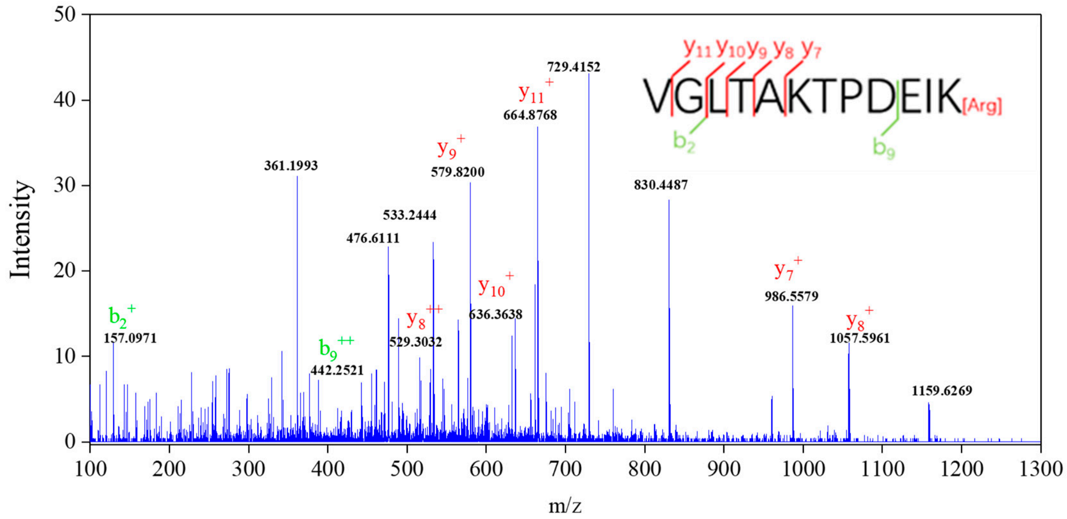
Intensity
m/z
VGLTAKTPDEIK[Arg]
y11 y10 y9 y8 y7
b2 b9
729.4152
y11+
664.8768
y9+
579.8200
361.1993
533.2444
476.6111
830.4487
y10+
636.3638
y8++
529.3032
b9++
442.2521
b2+
157.0971
y7+
986.5579
y8+
1057.5961
1159.6269

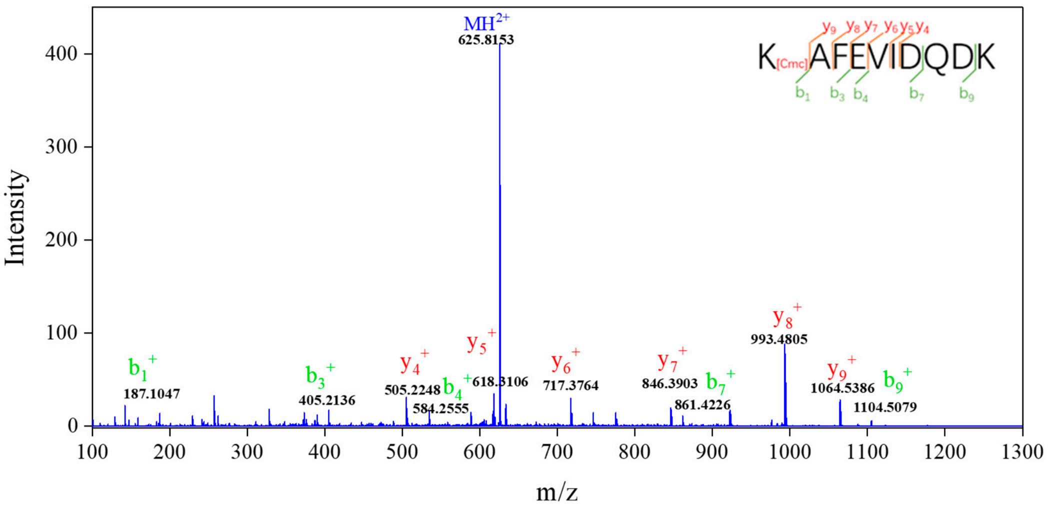
Intensity
m/z
K[Cmc]AFEVIDQDK
y9 y8 y7 y6 y5 y4
b1 b3 b4 b7 b9
MH2+
625.8153
b1+
187.1047
b3+
405.2136
y4+
505.2248
b4+
584.2555
y5+
618.3106
y6+
717.3764
y7+
846.3903
b7+
861.4226
y8+
993.4805
y9+
1064.5386
b9+
1104.5079

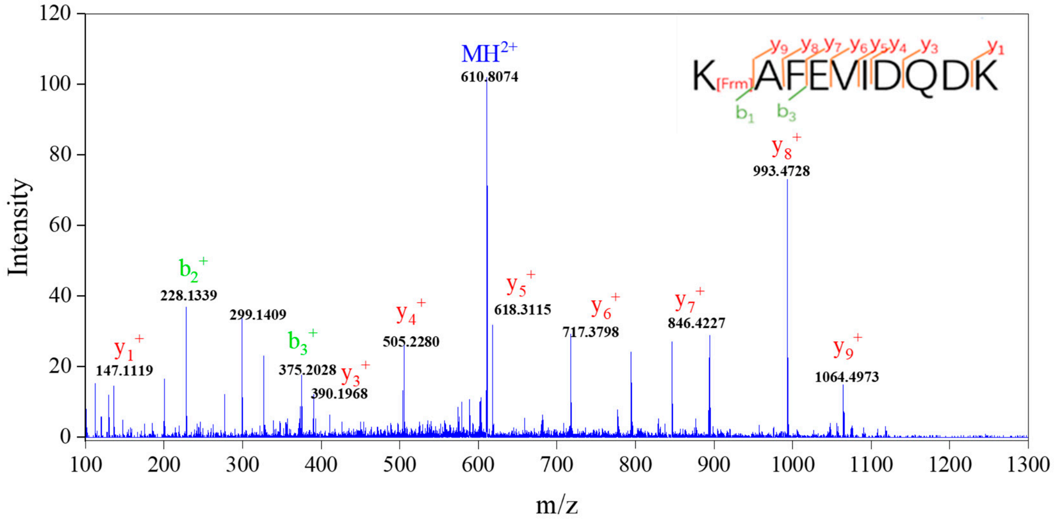
Intensity
m/z
MH2+
610.8074
K[Frm]AFEVIDQDK
y9 y8 y7 y6 y5 y4 y3 y1
b1 b3
y8+
993.4728
b2+
228.1339
299.1409
b3+
375.2028
y1+
147.1119
y3+
390.1968
y4+
505.2280
y5+
618.3115
y6+
717.3798
y7+
846.4227
y9+
1064.4973

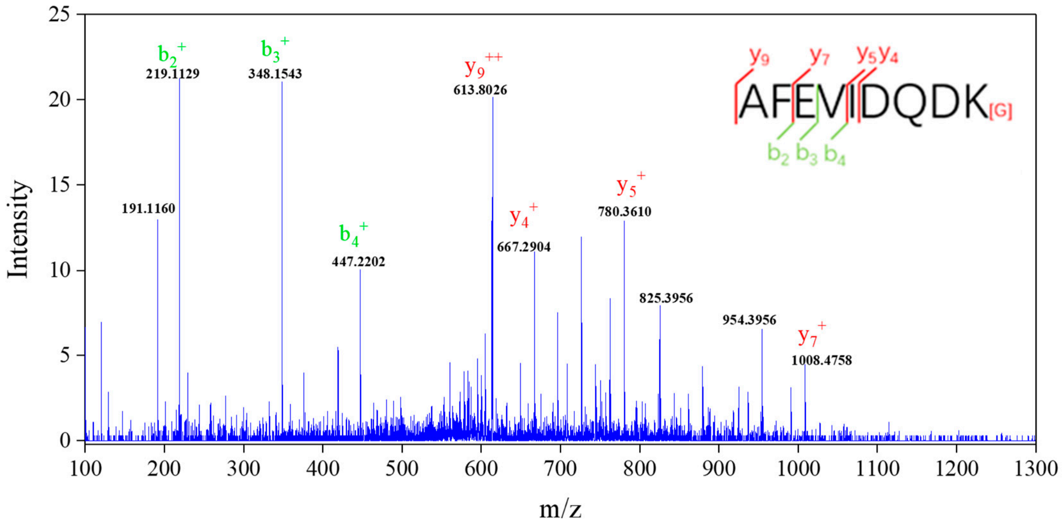
Intensity
m/z
AFEVIDQDK[G]
y9 y7 y5 y4
b2 b3 b4
b2+
219.1129
b3+
348.1543
y9++
613.8026
191.1160
b4+
447.2202
y4+
667.2904
y5+
780.3610
825.3956
954.3956
y7+
1008.4758

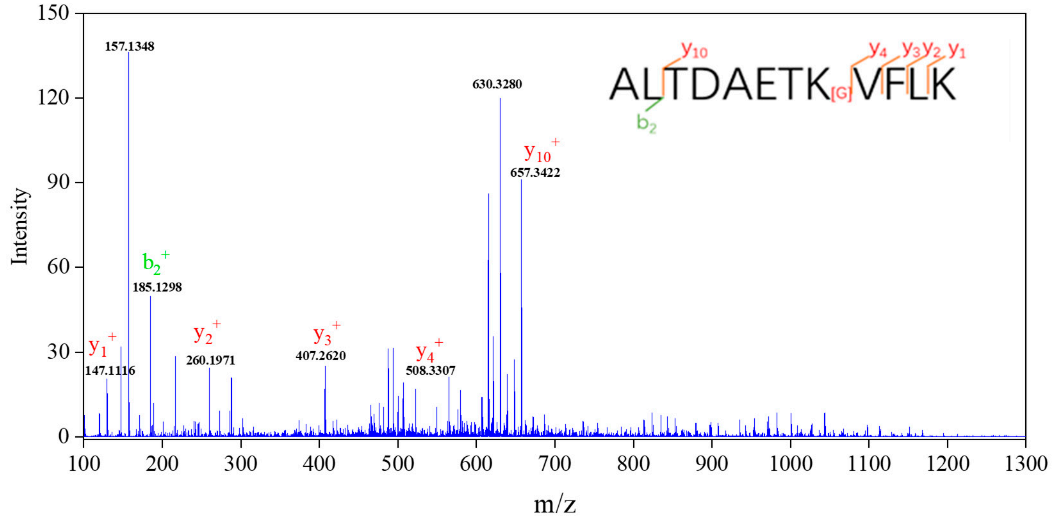
Intensity
m/z
ALTDAETK[G]VFLK
y10 y4 y3 y2 y1
b2
157.1348
630.3280
y10+
657.3422
b2+
185.1298
y1+
147.1116
y2+
260.1971
y3+
407.2620
y4+
508.3307

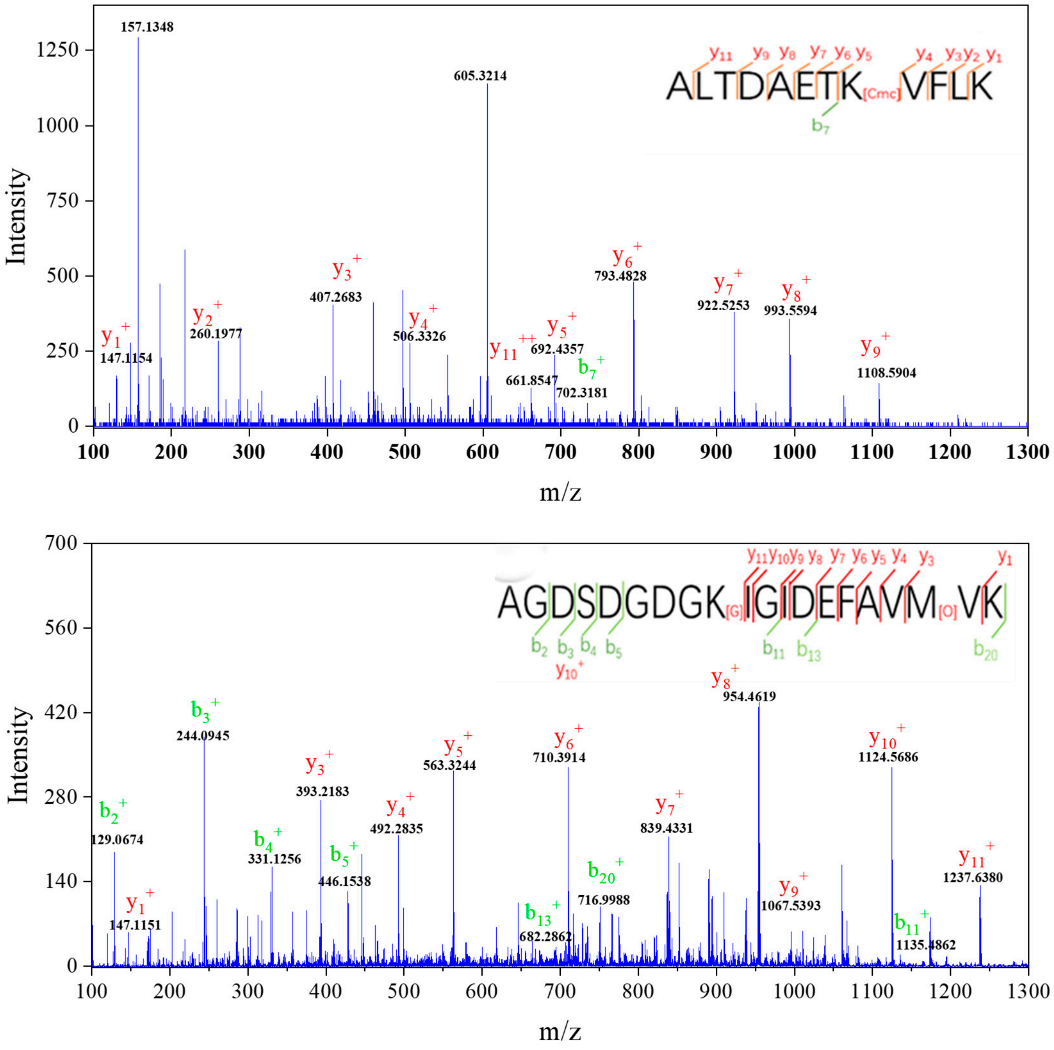


Figure S1. Identification of Maillard reaction modified PV-Ⅰ peptides in G3 using MS/MS.

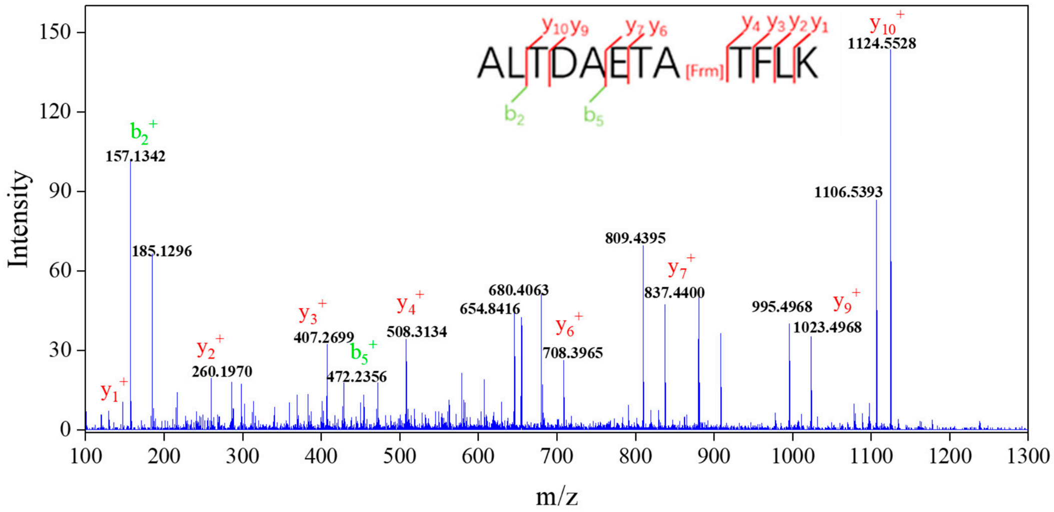


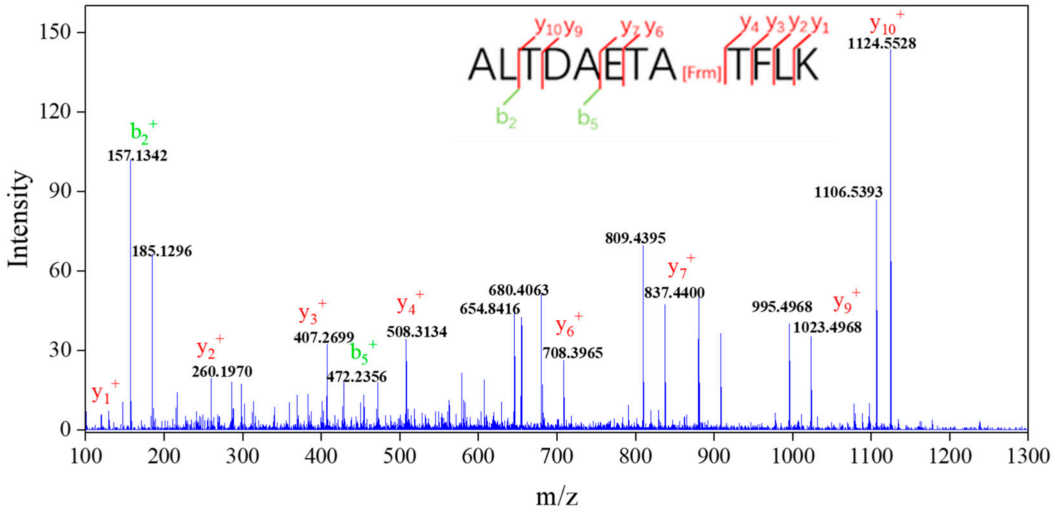


Figure S2. Identification of Maillard reaction modified PV-Ⅱ peptides in G3 using MS/MS.

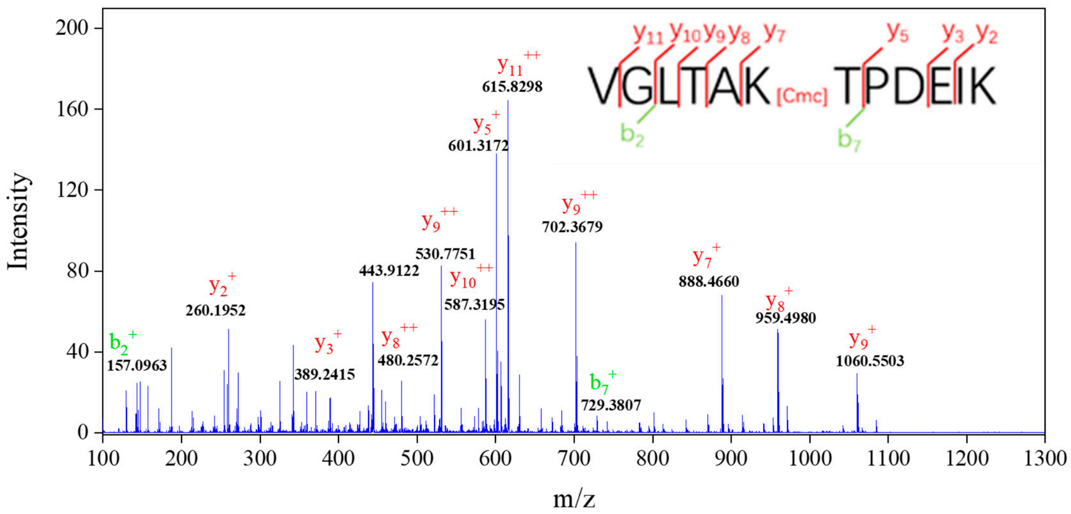
Intensity
m/z
VGLTAK[Cmc]TPDEIK
b2+ 157.0963
y2+ 260.1952
y3+ 389.2415
443.9122
y8++ 480.2572
y9++ 530.7751
y10++ 587.3195
y5+ 601.3172
y11++ 615.8298
y9++ 702.3679
b7+ 729.3807
y7+ 888.4660
y8+ 959.4980
y9+ 1060.5503

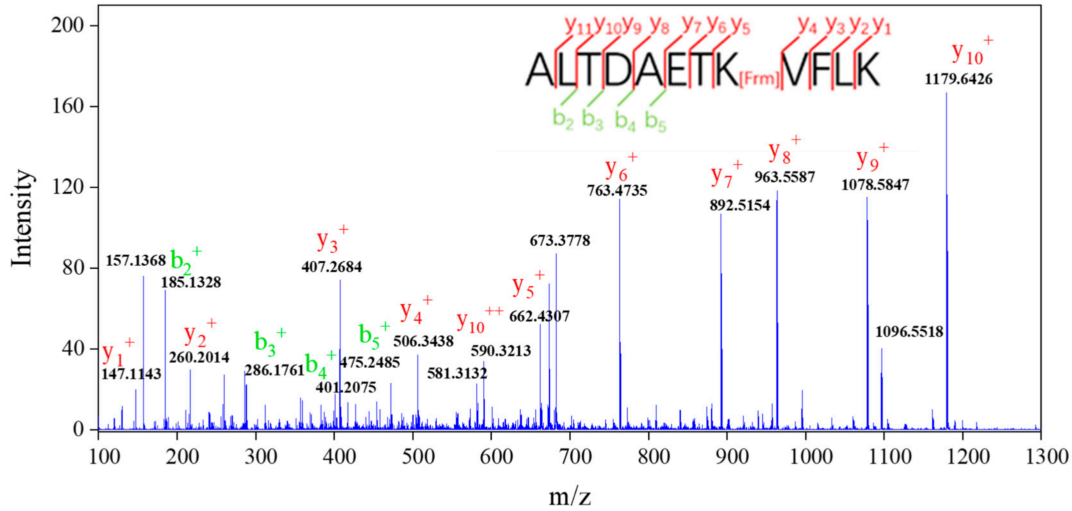
Intensity
m/z
ALTDAETK[Frm]VFLK
y1+ 147.1143
157.1368
b2+ 185.1328
y2+ 260.2014
b3+ 286.1761
b4+ 401.2075
y3+ 407.2684
b5+ 475.2485
y4+ 506.3438
581.3132
y10++ 590.3213
y5+ 662.4307
673.3778
y6+ 763.4735
y7+ 892.5154
y8+ 963.5587
y9+ 1078.5847
1096.5518
y10+ 1179.6426

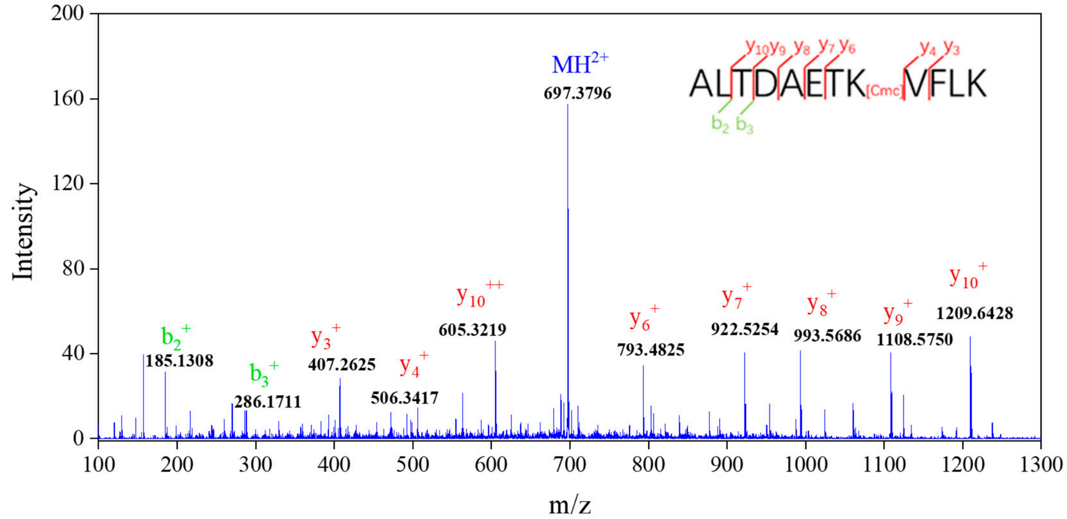
Intensity
m/z
ALTDAETK[Cmc]VFLK
MH2+ 697.3796
b2+ 185.1308
b3+ 286.1711
y3+ 407.2625
y4+ 506.3417
y10++ 605.3219
y6+ 793.4825
y7+ 922.5254
y8+ 993.5686
y9+ 1108.5750
y10+ 1209.6428

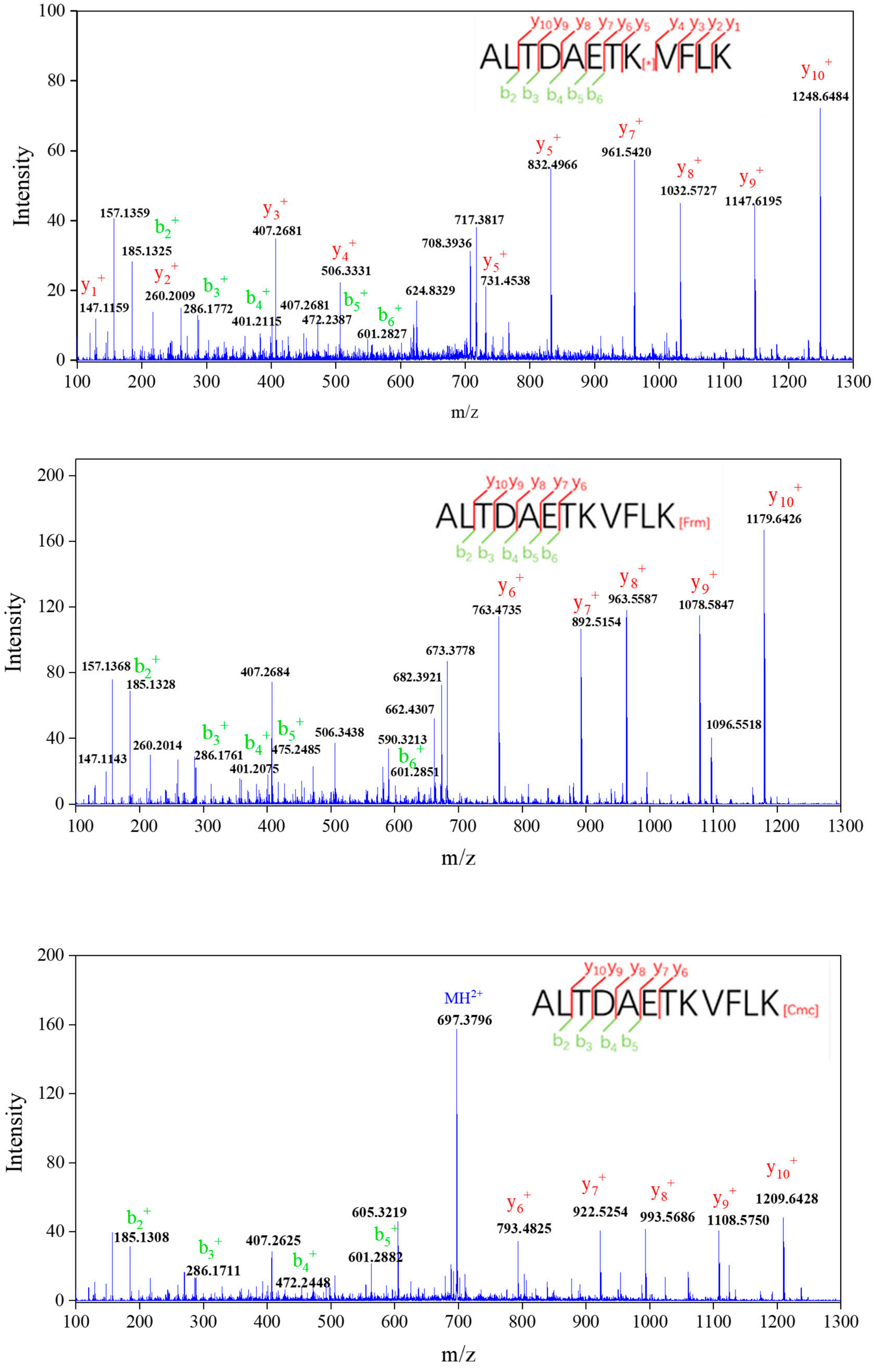


Figure S3. Identification of Maillard reaction modified PV-Ⅰ peptides in M2 using MS/MS.

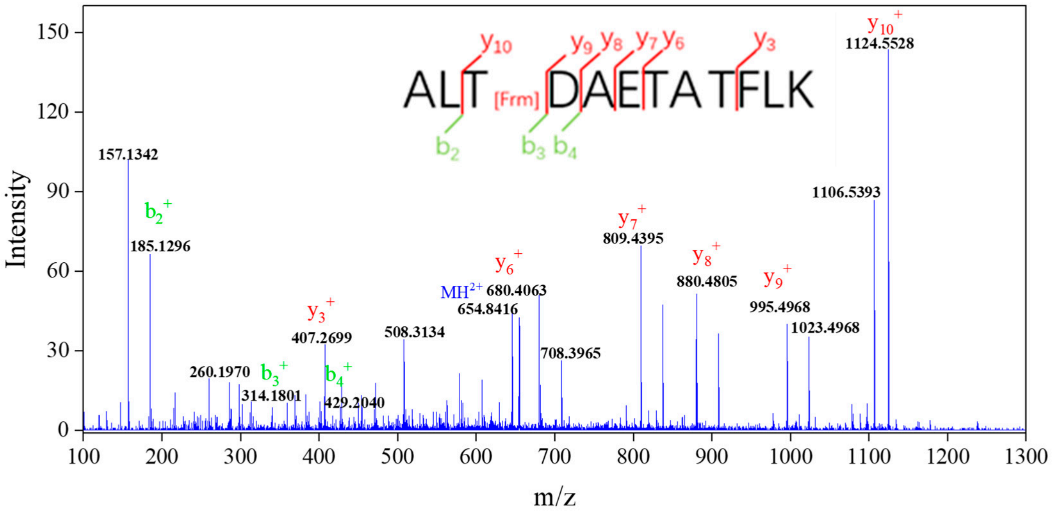


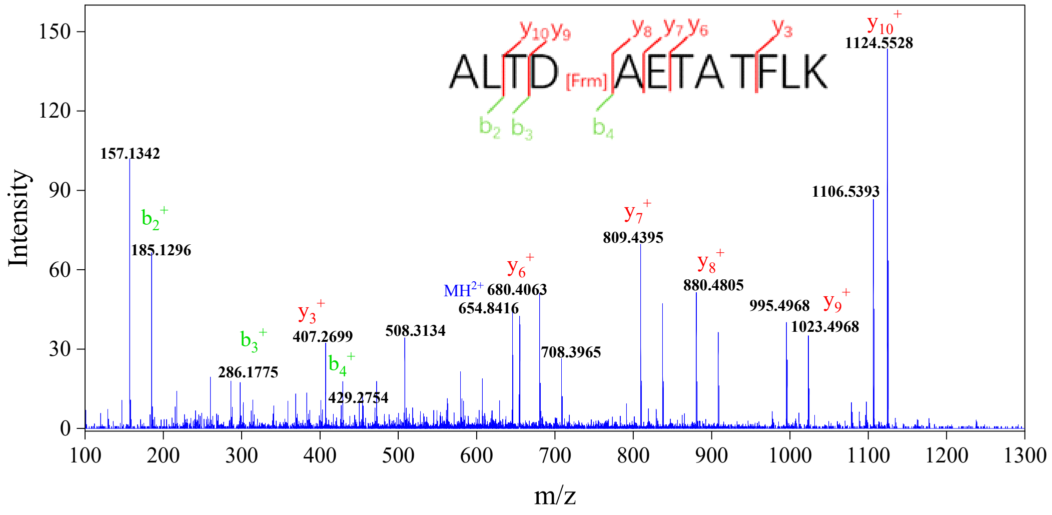


Figure S4. Identification of Maillard reaction modified PV-Ⅱ peptides in M2 using MS/MS.
